# Laparoscopic total pelvic exenteration for pelvic malignancies: the technique and short-time outcome of 11 cases

**DOI:** 10.1186/s12957-015-0715-2

**Published:** 2015-10-15

**Authors:** Kunlin Yang, Lin Cai, Lin Yao, Zheng Zhang, Cuijian Zhang, Xin Wang, Jianqiang Tang, Xuesong Li, Zhisong He, Liqun Zhou

**Affiliations:** Department of Urology, Peking University First Hospital, No. 8 Xishiku St, Xicheng District, Beijing, 100034 China; Institute of Urology, Peking University, No. 8 Xishiku St, Xicheng District, Beijing, 100034 China; National Urological Cancer Center, No. 8 Xishiku St, Xicheng District, Beijing, 100034 China; Department of General Surgery, Peking University First Hospital, No. 8 Xishiku St, Xicheng District, Beijing, 100034 China; Institute of General Surgery, Peking University First Hospital, No. 8 Xishiku St, Xicheng District, Beijing, 100034 China

**Keywords:** Laparoscopic, Total pelvic exenteration, Pelvic malignancy, Reconstruction

## Abstract

**Background:**

Previous reports about laparoscopic total pelvic exenteration (LTPE) are still limited. In the present study, we described our single-center experience of the initial 11 cases.

**Methods:**

Between April 2011 and September 2015, eight males and three females diagnosed as pelvic malignancies underwent LTPE by the same operation team. We retrospectively collected all cases’ parameters about surgical technique. Thirty-seven patients who received open surgery were also retrospectively collected. A comparison between LTPE and open surgery was performed to evaluate the feasibility and safety of LTPE.

**Results:**

Eleven cases successfully underwent the LTPE without any intraoperative complication. No open conversion was required. Eight patients underwent Bricker’s procedure. Three patients were performed with the cutaneous ureterostomy. Anus preservation operation was performed in three patients. Compared with open surgery, LTPE had longer mean operative time (565.2 vs 468.2 min, *p* = 0.004) but less mean blood loss (547.3 vs 1033.0 ml, *p* < 0.001) and shorter postoperative hospitalization time (15.3 vs 22.4 days, *p* = 0.004). One patient died of pulmonary embolism in the 7th month of follow-up time. One patient died of recurrence in the 12th month of follow-up time. Nine patients are still alive without recurrence and metastasis. The mean follow-up time was 11.1 months.

**Conclusions:**

The technique of LTPE seems to be feasible and safe in the treatment of carefully selected patients of pelvic malignancies. LTPE can also decrease the blood loss, the recovery time, and the hospital stay. But the oncological safety and long-term outcome of LTPE still need to be explored.

## Background

Total pelvic exenteration (TPE) is a surgical procedure that refers to a radical resection of the rectum, bladder, and reproductive organs. In 1948, it was first described by Brunschwig [1] as a palliative way for the terminal stages of the advanced pelvic malignancies. Classical open TPE has a high rate of postoperative morbidity but a relatively low mortality [2]. The open procedure is nowadays mainly used in the treatment of pelvic malignancies, such as locally advanced, recurrent cervical and colorectal cancer. It improves the long-term survival of patients with primary advanced rectal cancer [2].

In 2003, Pomel reported the first case of the laparoscopic total pelvic exenteration (LTPE) to treat the cervical cancer relapse [3]. From then, the LTPE was successively performed by some experienced laparoscopic centers. A cohort study has proved that the laparoscopic procedure is feasible and curative to selected patients [4]. From 2011, we began to carry out LTPE in our hospital by multidisciplinary cooperation.

We have searched for almost all literature about LTPE in the past decade. The articles about this procedure are still limited. In this study, we introduced our experience of LTPE with the initial eleven cases’ results.

## Methods

Eight males and three females were diagnosed as pelvic malignancies by biopsy and were selected to receive LTPE from April 2011 to September 2015. The selected criteria included the following: preoperative pathological diagnosis of pelvic malignancies (e.g., colorectal cancer, cervical cancer, or prostate cancer.), no evidence of distant metastasis, the possibility of complete resection, no surgical contraindication, and sufficient understanding about this procedure’s risk by the patient. The surgery was performed by the same surgical team. All cases’ demographic data, preoperative parameters (see Table 1), surgical parameters, and follow-up information about LTPE were retrospectively collected. The study was approved by the institutional review board from Peking University First Hospital.

**Table 1:** Patient demographics and preoperative parameters

Patient no.	Gender	Age	BMI	Preoperative diagnosis	Preoperative complications	nCRT	ASA
1	Male	57	25.3	Bladder transitional cell carcinoma (grade 3) and rectal adenocarcinoma	Lower gastrointestinal bleeding	No	2
2	Male	62	23.5	Prostate sarcoma (recurrent)	Difficult defecation	No	2
3	Male	58	19.9	Sigmoid adenocarcinoma (bladder invasion)	Rectovesical fistula Colonic obstruction	No	2
4	Male	62	23.3	Rectal adenocarcinoma (bladder invasion)	Rectovesical fistula	No	2
5	Male	75	25.9	Bladder transitional cell carcinoma (grade 2) and rectal adenocarcinoma	Acute renal insufficiency Hypertension	No	2
6	Female	69	21.7	Sigmoid adenocarcinoma (bladder and uterus invasion)	Hypertension	No	2
7	Female	55	23.4	Sigmoid adenocarcinoma (bladder invasion)	Rectovesical fistula	No	2
8	Male	44	24.7	Rectal adenocarcinoma (bladder invasion)	Renal calculi	No	2
9	Female	65	22.3	Sigmoid adenocarcinoma (bladder invasion)	Diabetes mellitus	No	2
10	Male	71	25.1	Sigmoid adenocarcinoma (bladder invasion)	Benign prostatic hyperplasia colonic obstruction	No	2
11	Male	30	20.2	Rectal adenocarcinoma (bladder invasion)	Rectal obstruction	No	2
Mean		58.9 (median, 62)	23.2				

*nCRT* neoadjuvant chemoradiotherapy, *ASA* American Standards Association, *BMI* body mass index

The following criteria for preoperative preparation were listed:

1. All patients had confirmed diagnosis with a preoperative biopsy.
2. Ultrasonography, enhanced computerized tomography scan or magnetic resonance imaging of the abdomen and pelvis, should be done to stage the disease and determine the extent of the tumor (see Fig. 2a–d, the bladder was invaded by tumor).
3. Preoperative standard bowel preparation in no ileus patient.
4. Evaluation of the physical condition to exclude any preoperative contraindication.
5. Informed contents were accepted and signed off by all patients and their family members.

To evaluate the efficiency and safety of LTPE, we also retrospectively collected the surgical parameters of 37 patients who received classical TPE from 2011 to 2015 to perform a comparison between LTPE and open surgery. The classical TPE needed a longitudinal incision (at least 15 cm) on the abdominal midline.

### Surgical technique

Epidural anesthesia was applied in combination with general anesthesia. All patients were equipped with patient-controlled analgesia after surgery.

**Fig. 1:**
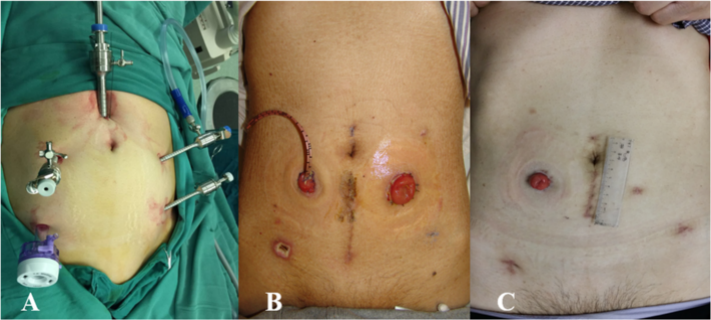
Trocar distribution and the situation of the stoma. **a** Trocar distribution; **b** the ileal bladder stoma and the sigmoid stoma of case 3; **c** the ileal bladder stoma of case 4 (the surgical scar is relatively small)

After anesthesia, patient was placed in Lloyd-Davis position. Pneumoperitoneum was established by open technique from the umbilicus. The positions of ports were modified from the Puntambekar’s way [5] (see Fig. 1a). A Trendelenburg (30°) position and right lateral tilt (30°) were maintained during the dissection of the sigmoid and rectum.

The procedure began with the dissection of posterior wall and lateral walls of the rectum. The right lateral peritoneum of the rectosigmoid was incised with the Harmonic Ace (Ethicon Endo-Surgery, Inc., Cincinnati, OH) by the middle enter approach. After entering to the posterior space of the rectosigmoid, the dissection was continued until to the root of inferior mesenteric artery. The inferior mesenteric vessels were ligated and cut. The left lateral peritoneum was also incised to meet with right lateral peritoneum. The retrorectal space was dissected to the level of levator ani muscle, and the lateral walls of the rectum were freed.

The tissues around the bladder and the ureter were dissected. Then, the bladder and the ureter were exposed. Seminiferous duct and superior vesical artery were bound with Hem-o-lok and cut with Harmonic Ace (Ethicon Endo-Surgery, Inc., Cincinnati, OH), following the bladder, lateral ligaments were cut with a Ligasure (Ligasure Vessel Sealing System: Valleylab, a division of Tyco Healthcare Group LP, Boulder, CO). The dissection should not be stopped until reaching the level of levator ani muscle. The urachus was cut off and the cave of Retzius was entered. The puboprostatic ligament was cut. After the dorsal vein complex was ligated and cut, the urethra and the ureter were cut with Harmonic Ace (Ethicon Endo-Surgery, Inc., Cincinnati, OH). The sigmoid was cut with the Endo-GIA.

**Fig. 2:**
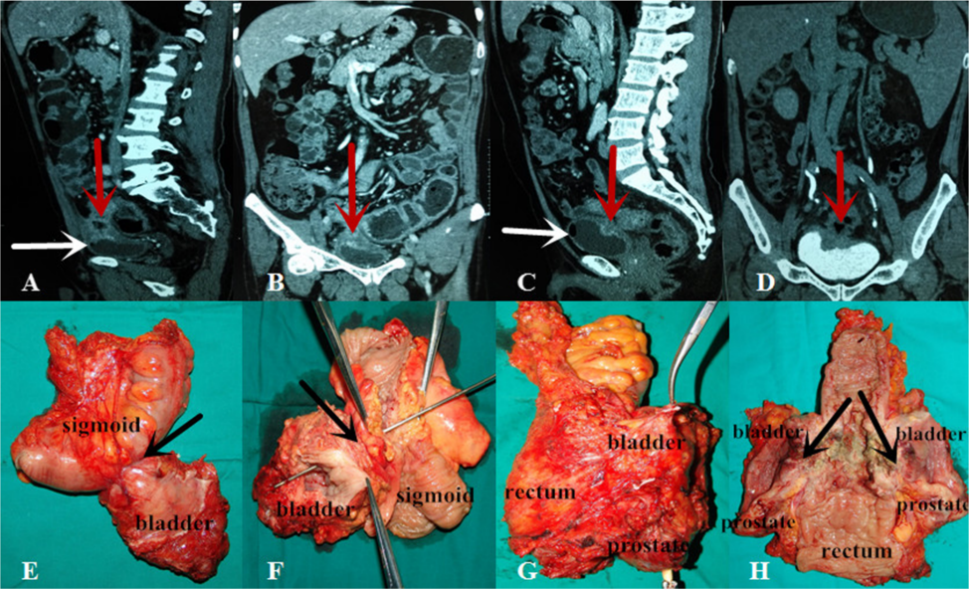
The computerized tomography (CT) image before surgery and surgical specimens of case 3 and case 4. **a**, **b** CT image of case 3, **c**, **d** CT image of case 4; the *red arrows* show the bladders are invaded by tumor; the *white arrows* show the air bubbles in the bladder because of rectovesical fistula; **e**, **f** the specimen of case 3, **g**, **h** the specimen of case 4 (the *black arrows* show the same results to the radiographic results)

Anus was sutured, and a new fusiform incision around the anus was performed. Ischiorectal fossa was dissected to the level of levator ani muscle. The specimen was removed (see Fig. 2e–h), and the fusiform incision was sutured. Further surgery needed a 4-cm vertical umbilical incision to perform the urinary diversion (like Bricker’s operation, cutaneous ureterostomy) and sigmoidostomy (see Fig. 1c).

If the patient was suitable for anus preservation operation, the operation was performed till the urethra and the ureter were cut according to the above steps. Then, the anterior rectal wall was exposed. After mesorectum was dissected, the rectum was cut with the Endo-GIA at about 5 cm away to the inferior margin of the tumor. A 4-cm vertical umbilical incision was performed. The bladder and rectal tumor was brought out from the incision, and the sigmoid was cut at about 10 cm away to the superior margin of the tumor. A string suture was performed at the end of the colon, and the anvil of a circular stapler was placed into it. The Bricker’s operation or cutaneous ureterostomy could be completed from the vertical incision (see Fig. 1b, c). Pneumoperitoneum was rebuilt after the incision was closed. At last, the head of the stapler was introduced from the anus into the distal stump. A colorectal anastomosis was successfully performed.

All patients routinely received the pelvic lymphadenectomy.

### Postoperative management

The drain was removed depending on the drainage volume. The stomas should be kept flowing well. The patients diagnosed with metastatic lymph nodes by the pathological examination all received the adjuvant chemotherapy.

## Results

Surgery was successfully performed in all patients. Eight patients underwent Bricker’s procedure after the bladder was resected. The cutaneous ureterostomy was performed in case 2, case 10, and case 11. Anus preservation operation was performed in case 4, case 7, and case 9. The details about operation parameters were shown in Table 2.

**Table 2:** Operative parameters and follow-up results

Patient no.	Radical	Construction type	Operative time/min	Blood loss/ml	Pathological stage	PRM	Postoperative hospitalization/day	Postoperative complication (<30 days after surgery)	Postoperative complication (>30 days after surgery)	Follow-up time/month	pCRT	Outcome
1	Yes	Bricker’s procedure and sigmoidostomy (Miles procedure)	620	500	Rectum pT2N0M0 and Bladder pT2bN0M0	Negative	23	No	DVT (7 months)	7	No	Died of non-oncological disease (PE)
2	Yes	Cutaneous ureterostomy and sigmoidostomy (Miles procedure)	415	600	Prostate pT4N0M0	Negative	10	No	UTI (6 months)	12	No	Died of oncological recurrence
3	Yes	Bricker’s procedure and sigmoidostomy (Hartmann procedure)	574	420	Sigmoid pT3 N1M0	Negative	15	No	No	24	XELOX	Alive
4	Yes	Bricker’s procedure and anus preservation operation	616	650	Rectum pT4b N1M0	Negative	11	No	No	22	XELOX	Alive
5	Yes	Bricker’s procedure and sigmoidostomy (Hartmann procedure)	690	800	Rectum pT3N0M0 Bladder pT1N0M0	Negative	26	No	No	14	No	Alive
6	Yes	Bricker’s procedure and sigmoidostomy (Hartmann procedure)	660	400	Sigmoid pT4b N1M0	Negative	14	No	No	14	XELOX	Alive
7	Yes	Bricker’s procedure and anus preservation operation	515	600	Sigmoid pT4b N0M0	Negative	9	No	No	10	XELOX	Alive
8	Yes	Bricker’s procedure and sigmoidostomy (Miles procedure)	520	850	Rectum pT4b N0M0	Negative	16	No	No	7	No	Alive
9	Yes	Bricker’s procedure and anus preservation operation	610	400	Sigmoid pT4b N0M0	Negative	13	No	No	5	No	Alive
10	Yes	Cutaneous ureterostomy and sigmoidostomy (Hartmann procedure)	450	200	Sigmoid pT4b N1M0	Negative	11	No	UTI	5	No	Alive
11	Yes	Cutaneous ureterostomy and sigmoidostomy (Miles procedure)	547	600	Rectum pT4b N1M0	Negative	20	Ileus	Ileus	2	XELOX	Alive
Mean			565.2	547.3			15.3			11.1		

*pCRT* postdischarge chemoradiotherapy, *XELOX* capecitabine + oxaliplatin, *PE* pulmonary embolism, *PRM* pathological resection margin, *DVT* deep vein thrombosis

The comparison between LTPE and open surgery was shown in Table 3. We could find that there were no differences in gender and age between two groups. But it was quite clear that LTPE had longer mean operative time (565.2 vs 468.2 min, *p* = 0.004), less mean blood loss (547.3 vs 1033.0 min, *p* < 0.001), and shorter mean postoperative hospitalization time (15.3 vs 22.4 days, *p* = 0.004).

**Table 3:** Comparison between LTPE and open surgery

	LTPE	Open surgery	*p*
Gender, (male/female)/n	8/3	20/17	0.60
Median age, range/year	62 (30–75)	55 (35–80)	0.53
Mean operative time ± SD, range/minute	565.2 ± 81.4 (415–690)	468.2 ± 51.8 (360–560)	0.004
Mean blood loss ± SD, range/ml	547.3 ± 180.1 (200–850)	1033.0 ± 284.6 (670–2000)	<0.001
Mean postoperative hospitalization ± SD, range/day	15.3 ± 5.3 (9–23)	22.4 ± 9.0 (10–45)	0.004

*LTPE* laparoscopic total pelvic exenteration, *SD* standard deviation

The mean follow-up time was 11.1 months. Case 1 died of pulmonary embolism in the 7th month of follow-up time. Case 2 died of oncological recurrence and metastasis in the 12th month of follow-up time. Case 3 to case 11 were still alive without indication of recurrence and metastasis when followed up. Case 11 suffered from ileus after surgery. After conservative treatment, the ileus was relieved without surgical intervention. Five patients received adjuvant chemotherapy of XELOX regiment (Oxaliplatin, 130 mg/m^2^, IV over 2 h, day 1 plus capecitabine, 850–1000 mg/m^2^, twice daily, PO, for 14 days; repeat every 3 weeks) 3 weeks after hospital discharge.

## Discussion

TPE has become a major surgical technique widely used for curative resection of locally advanced or recurrent pelvic malignancies since the 1940s. The classical TPE is open surgery which has a high rate of postoperative complication but relatively low surgical mortality. With the improvement of surgical techniques, the overall major morbidity rate after TPE is still up to 75 % (13 to 75 %) in previous literature [6–9], but the mortality has decreased from rates up to 33 % [10–14] down to rates less than 10 % (0 to 10 %) [15–17].

The emergence of laparoscopic surgery is an important milestone of the modern surgery. This revolution means the arrival of the minimal invasive surgery. Compared with open procedure, less intraoperative blood loss, less postoperative pain, and shorter hospital stay are the outstanding advantages of the laparoscopic procedure. Previously, the oncological outcome of the laparoscopic surgery was not acceptable, and the resection of the tumors was considered incomplete due to its small operative space. But in a randomized trail of 209 cases of colonic adenocarcinoma, the results showed that laparoscopy-assisted colectomy was more effective than open colectomy for treatment of colon cancer in terms of morbidity, hospital stay, tumor recurrence, and cancer-related survival [18]. Many other published studies have also proved the oncological safety of laparoscopic procedure [19–22]. With the advancement of technology and surgical skills, the laparoscopy is now widely used in the treatment of gynecological, colonic, and prostatic cancer.

In 2003, Pomel et al. reported the first case of LTPE with a cervical cancer relapse and showed the feasibility and safety of the procedure [3]. Subsequently, Lin et al. reported a case of laparoscopy-assisted transvaginal TPE [23]. In 2009, a robotic-assisted TPE was first reported by Peter in USA [24]. In 2011, an entirely robotic total pelvic exenteration and extended lymphadenectomy for recurrent endometrial cancer was reported by Vasilescu et al. [25]. Among the published literature, we found that 22 cases of different kinds of pelvic malignancies underwent LTPE in several oncological institutions around the world in the last decade (see Table 4) [3, 5, 23–31]. At present, there is still no large sample report about the long-term outcome of LTPE.

**Table 4:** Previous reports of laparoscopic total pelvic exenteration

Investigators	Year	Patient no.	Preoperative treatment	Mean operative time/min	Mean blood loss/ml	Type of UD	Conversion rate/%	Complication	Mean postoperative hospitalization/day	Follow-up time/mon	Follow-up outcome	5-year survival
Pomel et al. [3]	2003	1	Chemoradiotherapy	540	250	Bricker	0	0	16	NS	NS	NS
Lin et al. [23]	2004	1	Radiotherapy	540	200	US	0	UTI, SSI	19	12	Alive (disease free)	NS
Uzan et al. [24]	2005	2	Chemoradiotherapy	510 (480–540)	525 (250–800)	Bricker	0	UTI, CRAF	23.5 (17–30)	8.5 (6–11)	Dead	NS
Puntambekar et al. [25]	2006	2	NS	240	200	Wet colostomy	0	NS	3.5	15	NS	NS
[5]	2009	7	NS	230 (±15)	250 (±50)	Five wet colostomy, two Bricker	0	NS	8 (7–21)	11 (4–24)	Four died of distant metastases, three disease free more than a year	NS
Skrovina M et al. [26]	2006	3	1 NS 2 nCRT	NS	NS	Bricker	NS	One Wound dehiscence and AMI	NS	NS	NS	NS
Patel H et al. [27]	2009	2	Chemoradiotherapy	330	1200	Bricker	0	NS	11	NS	NS	NS
Lim PC [28] (robotic-assisted)	2009	1	Chemoradiotherapy	540	1000	Bricker	0	NS	23	NS	NS	NS
Figueiredo et al. [29]	2010	1	nCRT	450	NS	NS	NS	NS	NS	10	Alive (no evidence of recurrence and metastasis)	NS
Vasilescu et al. [30] (entirely robotic)	2011	1	Radiotherapy	250	365	Cutaneous ureterostomy	0	0	11	NS	NS	NS
Mukai et al. [31]	2013	1	nCRT	831	600	Cutaneous ureterostomy	0	Ileus	29	NS	NS	NS
Total		22										

*UD* urinary diversion, *NS* not stated, *UTI* urinary tract infection, *SSI* surgical site infection, *US* ureterosigmoidostomy, *CRAF* colorectal anastomosis fistula, *nCRT* neoadjuvant chemoradiotherapy, *AMI* acute myocardial infarction

Previous reports show that mean operative time of LTPE is ranging from 230 up to 831 min, and mean postoperative hospitalization stay varies from 3.5 to 29 days. In our series, mean operative time and mean hospital stay are similar to others, respectively 565.2 min and 15.3 days. But both parameters are much shorter in Indian study than other studies. It may be the result of different surgical techniques and different health policies. In addition, all the cases were female patients whose anatomy is relatively simple in Indian study. The hospital stay is also closely associated with the postoperative complications. The mean estimated blood loss of our study is obviously reduced when patients underwent laparoscopic approach, ranging from 200 to 850 ml, compared to classical open approach, ranging from 1000 to 7550 ml [6, 32].

TPE is one of the most spoiling surgeries, requiring en bloc resection of the pelvic organs. More than half of the patients underwent the TPE suffered from different kinds of major or minor complications associated with urinary diversion and bowel reconstruction [33], especially previous radiotherapy before surgery [34]. We hope that the laparoscopic approach can play an important role in reducing the morbidity rate. But dramatically, in a cohort study, it did not work when compared to open approach [4]. Few data were reported. More scientific studies are still needed.

The most commonly used form of urinary diversion (UD) for pelvic exentenration is Bricker’s ileal conduit (IC) described first in 1950 [35]. IC has been the safest and easiest way for urinary diversion while decreasing the morbidity rate. The advantage of this form is to overcome the high complication rate caused by primary wet colostomy. Primary wet colostomy has been abandoned because of the 9 % high mortality rate of complications [36], like electrolyte abnormalities, ascending pyelonephritis, and malodorous watery diarrhea. In 1989, Carter et al. [37] first described a modified wet colostomy technique called double-barreled wet colostomy (DBWC). In 2010, Golda et al. [38] reported their single institution experience about DBWC drawing a conclusion that DBWC is an alternative option for patients after TPE when reconstruction of the fecal and urinary streams is not possible. Compared with traditional IC, DBWC provides a single stoma allowing for easier maintenance and not increasing the morbidity rates. Chokshi et al. [39] concluded the similar result with Golda’s study that DBWC is able to provide a safe and feasible technique for urinary and fecal diversion.

In 11 cases of our study, eight patients underwent Bricker’s IC and three patients received cutaneous ureterostomy (CU). Because three patients were terminal, CU was performed as a temporary diversion. We can find that the operative time of case 2 and case 10 was obviously much shorter than others. The procedure of CU is simple and no need for bowel resection and anastomosis. CU is just widely used for abdominal wall diversion in children but rarely done in adult. The procedure is only used for diversion in terminal stage or when bowel resection cannot be performed [40].

For fecal diversion, eight patients underwent the sigmoidostomy and three patients underwent anus preservation operation. In case 1, the distance of rectal tumor from the anus verge was 3 cm, so a sigmoidostomy (Miles procedure) was performed. But in case 4, patient received the anus preservation operation as the distance of tumor from the anus verge was 10 cm (>5 cm). This situation was similar to case 7 and case 9. For case 2, case 3, case 5, case 6, and case 10, Hartmann’s procedure was performed in consideration of the possible subsequent radiotherapy or chemotherapy and the high risk of bowel anastomosis’ leakage.

Traditionally, open TPE is associated with high complications rates of UD and fecal diversion. Complication rate directly related to UD after laparoscopic approach is reported more than 50 % [4]. According to both open approach and laparoscopic approach, the most common complication is infection, especially urinary tract infection (UTI, 21–36 %) [4], followed by ureteral stricture (5–22.1 %) [4], ureteral/anastomosis leaks (8–14 %) [4], urinary stomal stricture (4–25 %) [4], and stone formation (2–18 %) [4]. In our study, UTI occurred in two patients (2/7, 28.6 %) more than 30 days after operation. Deep vein thrombosis (DVT) occurred in one patient, and the patient died of pulmonary embolism; the others were well. The patient suffered increased stool frequency at 2 months after anus preservation operation. It was still unknown whether LTPE can really reduce the high morbidity rates or not. More contrast studies about morbidity rate of TPE between open approach and laparoscopic approach need to be done. It is proved, in this study, that the laparoscopic approach is advantageous in decreasing the recovery time, blood loss, and hospital stay.

In this study, all patients did not received the preoperative chemotherapy and radiotherapy. For case 3, case 4, case 6, and case 11, because the pathologic staging information showed positive regional lymph nodes, XELOX regimen as postoperative chemotherapy was recommended to patients. Case 10 refused to accept postoperative chemotherapy. No complications-related chemotherapy occurred. By now, no evidences of recurrence and metastasis have been found in these patients.

In reported literature, more than half of cases were females who underwent the LTPE because of cervical cancer. But in our study, only three cases were females. Because the physiology and pelvic anatomy of the male are different from those of the female, we do not know whether the gender is an influencing factor to the LTPE or not. Larger sample and gender-related analysis will be needed in further study.

Although the oncological safety of the laparoscopic approach has been accepted in treating many oncological diseases, the oncological safety of LTPE is still indeterminate as follow-up time was very limited; no series reach 5 years of follow-up time.

We believe that the selection of patients is crucial for a satisfactory oncological result. Patients’ compliance and patients’ education regarding the LTPE are also important. The patient should know this surgical approach means the obvious decrease of the quality of life. The multidisciplinary team is an important guaranty of the successful LTPE. Based on our experiences, at least the urologist, the colorectal specialist and special postoperative nursing group are needed.

Although in our study, the number of cases is maybe the largest compared with previous published articles, 11 was far from enough for a persuasive study. In addition, this was a retrospective study which was another limitation. A randomized control trial will be needed in our later work.

## Conclusions

In conclusion, with the initial experience of 11 cases of LTPE, we think that the technique of LTPE seems to be a feasible and safe procedure in the treatment of carefully selected patients of pelvic malignancies. LTPE is also advantageous in decreasing blood loss, recovery time, and hospital stay. But the oncological safety and long-term outcome of LTPE still need to be explored in the future.

## Consent

Written informed consent was obtained from the patient for the publication of this report and any accompanying images.

## Abbreviations

LTPE: laparoscopic total pelvic exenteration
TPE: total pelvic exenteration
UD: urinary diversion
IC: ileal conduit
DBWC: double-barreled wet colostomy
CU: cutaneous ureterostomy
UTI: urinary tract infection
DVT: deep vein thrombosis

## References

[1] Brunschwig A (1948). Complete excision of pelvic viscera for advanced carcinoma; a one-stage abdominoperineal operation with end colostomy and bilateral ureteral implantation into the colon above the colostomy. Cancer.

[2] Nielsen MB, Rasmussen PC, Lindegaard JC, Laurberg S (2012). A 10-year experience of total pelvic exenteration for primary advanced and locally recurrent rectal cancer based on a prospective database. Colorectal Dis.

[3] Pomel C, Rouzier R, Pocard M, Thoury A, Sideris L, Morice P (2003). Laparoscopic total pelvic exenteration for cervical cancer relapse. Gynecol Oncol.

[4] Martinez A, Filleron T, Vitse L, Querleu D, Mery E, Balague G (2011). Laparoscopic pelvic exenteration for gynaecological malignancy: is there any advantage?. Gynecol Oncol.

[5] Puntambekar SP, Agarwal GA, Puntambekar SS, Sathe RM, Patil AM (2009). Stretching the limits of laparoscopy in gynecological oncology: technical feasibility of doing a laparoscopic total pelvic exenteration for palliation in advanced cervical cancer. Int J Biomed Sci.

[6] Vermaas M, Ferenschild FT, Verhoef C, Nuyttens JJ, Marinelli AW, Wiggers T (2007). Total pelvic exenteration for primary locally advanced and locally recurrent rectal cancer. Eur J Surg Oncol.

[7] Saito N, Koda K, Takiguchi N, Oda K, Ono M, Sugito M (2003). Curative surgery for local pelvic recurrence of rectal cancer. Dig Surg.

[8] Law WL, Chu KW, Choi HK (2000). Total pelvic exenteration for locally advanced rectal cancer. J Am Coll Surg.

[9] Yamada K, Ishizawa T, Niwa K, Chuman Y, Aikou T (2002). Pelvic exenteration and sacral resection for locally advanced primary and recurrent rectal cancer. Dis Colon Rectum.

[10] Lindsey WF, Wood DK, Briele HA, Greager JA, Walker MJ, Bork J (1985). Pelvic exenteration. J Surg Oncol.

[11] Lopez MJ, Standiford SB, Skibba JL (1994). Total pelvic exenteration. A 50-year experience at the Ellis Fischel Cancer Center. Arch Surg.

[12] Boey J, Wong J, Ong GB (1982). Pelvic exenteration for locally advanced colorectal carcinoma. Ann Surg.

[13] Falk RE, Moffat FL, Makowka L, Konn G, Bulbul MA, Rotstein LE (1985). Pelvic exenteration for advanced primary and recurrent adenocarcinoma. Can J Surg.

[14] Shirouzu K, Isomoto H, Morodomi T, Ogata Y, Akagi Y, Kakegawa T (1995). Total pelvic exenteration for locally advanced colorectal carcinoma—postoperative complications. Kurume Med J.

[15] Hafner GH, Herrera L, Petrelli NJ (1992). Morbidity and mortality after pelvic exenteration for colorectal adenocarcinoma. Ann Surg.

[16] Lopez MJ, Monafo WW (1993). Role of extended resection in the initial treatment of locally advanced colorectal carcinoma. Surgery.

[17] Liu SY, Wang YN, Zhu WQ, Gu WL, Fu H (1994). Total pelvic exenteration for locally advanced rectal carcinoma. Dis Colon Rectum.

[18] Lacy AM, Garcia-Valdecasas JC, Delgado S, Castells A, Taura P, Pique JM (2002). Laparoscopy-assisted colectomy versus open colectomy for treatment of non-metastatic colon cancer: a randomised trial. Lancet.

[19] A comparison of laparoscopically assisted and open colectomy for colon cancer. N Engl J Med. 2004;350:2050–9. 10.1056/NEJMoa032651 15141043

[20] Di B, Li Y, Wei K, Xiao X, Shi J, Zhang Y (2013). Laparoscopic versus open surgery for colon cancer: a meta-analysis of 5-year follow-up outcomes. Surg Oncol.

[21] Liang Y, Li G, Chen P, Yu J (2008). Laparoscopic versus open colorectal resection for cancer: a meta-analysis of results of randomized controlled trials on recurrence. Eur J Surg Oncol.

[22] Van der Pas MH, Haglind E, Cuesta MA, Furst A, Lacy AM, Hop WC (2013). Laparoscopic versus open surgery for rectal cancer (COLOR II): short-term outcomes of a randomised, phase 3 trial. Lancet Oncol.

[23] Lin MY, Fan EW, Chiu AW, Tian YF, Wu MP, Liao AC (2004). Laparoscopy-assisted transvaginal total exenteration for locally advanced cervical cancer with bladder invasion after radiotherapy. J Endourol.

[24] Uzan C, Rouzier R, Castaigne D, Pomel C (2006). Laparoscopic pelvic exenteration for cervical cancer relapse: preliminary study. J Gynecol Obstet Biol Reprod (Paris).

[25] Puntambekar S, Kudchadkar RJ, Gurjar AM, Sathe RM, Chaudhari YC, Agarwal GA (2006). Laparoscopic pelvic exenteration for advanced pelvic cancers: a review of 16 cases. Gynecol Oncol.

[26] Bartos P, Skrovina M, Trhlik M (2009). Videopresentation of laparoscopic technique of anterior, posterior and total exenteration for recurrent cervical cancer. Int J Gynecol Obstet.

[27] Patel H, Joseph JV, Amodeo A, Kothari K (2009). Laparoscopic salvage total pelvic exenteration: is it possible post-chemo-radiotherapy?. J Minim Access Surg.

[28] Lim PC (2009). Robotic assisted total pelvic exenteration: a case report. Gynecol Oncol.

[29] Figueiredo JA, Carvalho GM, Mota RT, Castro VM (2011). Meyer MMMDE, Barragat AZ. Laparoscopic total pelvic exenteration and perineal amputation with wet colostomy. A case report. J Coloproctol.

[30] Vasilescu C, Tudor S, Popa M, Aldea B, Gluck G (2011). Entirely robotic total pelvic exenteration. Surg Laparosc Endosc Percutan Tech.

[31] Mukai T, Akiyoshi T, Ueno M, Fukunaga Y, Nagayama S, Fujimoto Y (2013). Laparoscopic total pelvic exenteration with en bloc lateral lymph node dissection after neoadjuvant chemoradiotherapy for advanced primary rectal cancer. Asian J Endosc Surg.

[32] Maggioni A, Roviglione G, Landoni F, Zanagnolo V, Peiretti M, Colombo N (2009). Pelvic exenteration: ten-year experience at the European Institute of Oncology in Milan. Gynecol Oncol.

[33] Diver EJ, Rauh-Hain JA, Del CM (2012). Total pelvic exenteration for gynecologic malignancies. Int J Surg Oncol.

[34] Houvenaeghel G, Moutardier V, Karsenty G, Bladou F, Lelong B, Buttarelli M (2004). Major complications of urinary diversion after pelvic exenteration for gynecologic malignancies: a 23-year mono-institutional experience in 124 patients. Gynecol Oncol.

[35] Bricker EM (1950). Bladder substitution after pelvic evisceration. Surg Clin North Am.

[36] Brunschwig A, Pierce VK (1950). Partial and complete pelvic exenteration; a progress report based upon the first 100 operations. Cancer.

[37] Carter MF, Dalton DP, Garnett JE (1989). Simultaneous diversion of the urinary and fecal streams utilizing a single abdominal stoma: the double-barreled wet colostomy. J Urol.

[38] Golda T, Biondo S, Kreisler E, Frago R, Fraccalvieri D, Millan M (2010). Follow-up of double-barreled wet colostomy after pelvic exenteration at a single institution. Dis Colon Rectum.

[39] Chokshi RJ, Kuhrt MP, Schmidt C, Arrese D, Routt M, Parks L (2011). Single institution experience comparing double-barreled wet colostomy to ileal conduit for urinary and fecal diversion. Urology.

[40] Rodriguez AR, Lockhart A, King J, Wiegand L, Carrion R, Ordorica R (2011). Cutaneous ureterostomy technique for adults and effects of ureteral stenting: an alternative to the ileal conduit. J Urol.

